# Early Nuclear Events after Herpesviral Infection

**DOI:** 10.3390/jcm8091408

**Published:** 2019-09-07

**Authors:** Florian Full, Armin Ensser

**Affiliations:** Institute for Clinical and Molecular Virology, University Hospital Erlangen, Friedrich Alexander University Erlangen-Nuremberg, 91054 Erlangen, Germany

**Keywords:** Herpesviruses, double-stranded DNA virus, DNA-damage repair, DNA-damage response, nuclear DNA sensors, restriction factors, HSV-1, Herpes simplex virus, CMV, cytomegalovirus, KSHV, Kaposi’s sarcoma-associated herpesvirus, double strand break, NHEJ, non-homologous end joining, HR, homology repair, infection, virus, PML, PML nuclear bodies, ND10, virus-host interaction, SP100, DAXX, ATRX, ICP-0, IE-1, ORF75, HHV-6

## Abstract

Herpesviruses are important pathogens that can cause significant morbidity and mortality in the human population. Herpesviruses have a double-stranded DNA genome, and viral genome replication takes place inside the nucleus. Upon entering the nucleus, herpesviruses have to overcome the obstacle of cellular proteins in order to enable viral gene expression and genome replication. In this review, we want to highlight cellular proteins that sense incoming viral genomes of the DNA-damage repair (DDR) pathway and of PML-nuclear bodies (PML-NBs) that all can act as antiviral restriction factors within the first hours after the viral genome is released into the nucleus. We show the function and significance of both nuclear DNA sensors, the DDR and PML-NBs, and demonstrate for three human herpesviruses of the alpha-, beta- and gamma-subfamilies, HSV-1, HCMV and KSHV respectively, how viral tegument proteins antagonize these pathways.

## 1. Introduction

Herpesviruses have evolved sophisticated ways to subvert the immune system over millions of years of coevolution with their respective hosts. While herpesviruses also need to surmount adaptive cell-mediated immunity in order to prevent being killed by immune cells and establish persistent infection, the antagonism of cellular restriction and the innate immune system is of particular importance in order to achieve efficient infection of target cells. The nine human members of the herpesvirus family include important human pathogens, i.e., Herpes-simplex viruses-1 and -2 (HSV-1 and HSV-2), the Varicella-Zoster Virus (VZV), Human Cytomegalovirus (HCMV), Human Herpesviruses-6A, -6B and -7, and the tumor viruses Epstein-Barr virus (EBV) and Kaposi’s sarcoma associated herpesvirus (KSHV) [1]. A hallmark of herpesvirus infections is the establishment of lifelong latency [1]; for example, about 3.7 billion people are infected with HSV-1 worldwide [2], and even more by HHV-6, -7, and EBV [3,4]. In this regard, herpesviruses are the most “successful” virus family in the human population, because almost all human adults are latently infected with at least one herpesvirus, and most by several. Herpesviruses are double-stranded DNA viruses with genome sizes from 125–250 kbp [1]. For the infection of target cells, herpesviral virus particles bind to cellular surface receptors. Virus particles are internalized, by fusion or endocytosis, the capsids are transported to the nucleus, and the linear genome is released into the nucleus through nuclear pores. Whereas the viral genome is enclosed and protected by capsid proteins in the cytoplasm, upon injection into the nucleus, the naked linear viral DNA is especially vulnerable to detection by cellular DNA-damage proteins and to attack by cellular restriction factors. Therefore, the viruses had to evolve ways to (i) prevent detection of the genome by DNA-damage proteins; (ii) prevent degradation of the naked, linear viral DNA; (iii) realize recircularization of the viral genome; (iv) achieve chromatinization of the genome, but simultaneously, prevent the addition of repressive chromatin marks, and (v) establish efficient viral gene expression. In our review, we want to focus on the nuclear events of herpesviral infection in the first hours after the viral genome enters the nucleus by focusing on one human member of each of the alpha-, beta-, and gamma-herpesviruses, namely HSV-1, HCMV, and KSHV. We try to highlight the critical steps and their importance for the herpesviral life cycle, and show similarities and differences between herpesvirus subfamilies. In addition, we want to demonstrate how herpesviral proteins, in particular tegument proteins that enter the cell as part of the viral particle, antagonize cellular restriction factors.

## 2. DNA Sensor Proteins

Upon infection, the viral capsid is transported through the cytoplasm along the cytoskeleton until it reaches the nuclear membrane; there, the viral genome, which is coiled in the capsid under high pressure, is injected through nuclear pores [5]. In the cytoplasm, sensing of viral DNA by DNA-sensors like AIM2 and cGAS leads to the activation of both the STING-IRF3-NF-kB pathway and the inflammasome; this results in antiviral cytokine production, including interferon and interferon-stimulated genes [6,7,8]. AIM2, cGAS, and STING are predominantly located in the cytoplasm, which has prompted controversy about the mechanisms of herpesviral DNA-sensing [9]. Given the model of infection described above, capsid proteins always protect the viral DNA, and there is no naked viral DNA present in the cytoplasm during the natural way of infection [5,10]. However, at least after replication in cell culture, the majority of herpesviral particles are not able to establish infection, as reflected by (packaged genome copies: plaque forming unit) ratios of >10–1000. In addition, there are also many empty capsids and virions present. Thus, it is conceivable that there is sensing of such defective viral particles that may contain or spill accessible and unprotected viral DNA. The uptake of such defective particles by cells during infection may initiate cGAS- and STING-dependent signaling events that elicit a potent type-I IFN response. In addition, the cellular protein IFI16 has also been shown to act as a sensor for herpesviral DNA [11,12,13]. Most work on IFI16 as an antiviral sensor was done with HSV-1, and the data about the antiviral role was partially controversial, in particular with respect to the effect of the depletion of IFI16 on herpesviral replication and the role of ICP0 in the degradation of IFI16 [14]. Nevertheless, it is well accepted that IFI16 has an important function in sensing herpesviral DNA [9]. In contrast to cGAS and STING, IFI16 is a predominantly nuclear protein [11,12], and most publications point at nuclear sensing of herpesviral DNA by IFI16 [14]. In the initial publication describing the antiviral role of IFI16, it was proposed that IFI16 could shuttle between the nucleus and the cytoplasm to initiate innate immune signaling [15]. IFI16 can be acetylated within its nuclear localization signal, which results in the translocation of the protein to the cytoplasm [13]; for KSHV, this acetylation, followed by translocation, might be involved in sensing the virus [16]. However, it was also shown that a fraction of cGAS can locate to the nucleus in primary cells, and that cGAS stabilizes IFI16, and thereby promotes, the sensing of HSV-1 [17]. Moreover, several reports suggest that IFI16 could be directly associated with herpesviral DNA in the nucleus [16,18], most likely mediated through oligomerization via its Pyrin domains [19]. As a consequence of oligomerization, IFI16 can form filamentous structures in viral replication compartments after infection with an ICP0-deficient HSV-1, and it is thought that these structures restrict viral gene expression [20]. For HCMV, the knockdown of IFI16 has been shown to facilitate viral replication [21], and HCMV also encodes for a protein, pUL83 (pp65), that prevents oligomerization of IFI16 via its Pyrin domains, and thereby inhibits innate immune activation [22]. A recombinant HCMV with a deletion of the pp65 gene showed an increase in IFN-beta production compared to wildtype HCMV, which illustrates the importance of IFI16 in the innate immune response to herpesviral infection. In contrast to HCMV, HSV-1 infection results in the degradation of IFI16. It is unclear whether this degradation is mediated by ICP0 [14]. While some publications show that the degradation of IFI16 is mediated by ICP0 [12], others show that this degradation is independent of ICP0 [23], and that ICP0 even prevents the association of IFI16 with viral DNA [23,24]. Most likely, the observed differences can be explained by cell type-specific regulation of IFI16 in response to HSV-1 infection [25]. Similar to HSV-1, degradation of IFI16 has also been shown during the lytic reactivation of KSHV [26] as an immune evasion mechanism to prevent inflammasome activation [27]. KSHV infection leads to inflammasome activation through a unique mechanism that includes IFI16 and BRCA1 translocation into the cytoplasm [28]. In addition to BRCA1, IFI16 also recruits histone H2B and the acetyl-transferase p300 to sites of viral genomes [29]. Subsequently, H2B and IFI16 get acetylated by p300, which leads to translocation of the entire IFI16-BRCA1-p300-H2B complex into the cytoplasm, followed by inflammasome activation and Il1-beta secretion [29]. Mechanistically, the direct restriction of gene expression by IFI16 could be explained by the introduction of repressive histone modifications on viral chromatin, as has been reported for HSV-1 [11,30]. More studies are needed to identify the cellular enzymes that mediate respective histone modifications and whether they are directly recruited by IFI16 to viral genomes. IFI16 is rapidly recruited to incoming viral genomes [18,22], and several studies show IFI16 oligomerization in the proximity of viral genomes at later time points using confocal and live cell microscopy [19,31]. Intriguingly, there was no evidence for the association of viral DNA with IFI16 or PML-NBs after nuclear entry of adenoviruses [32,33]. It could be that there is a fundamental difference in the recognition of herpesviruses and adenoviruses by IFI16. In addition, the detection of incoming viral genomes is technically challenging, which makes it difficult to perform in-depth biochemical analyses. Nevertheless, it was demonstrated by confocal microscopy in a recent study by the Knipe lab that IFI16 and ATRX independently localize to EdC-labelled HSV-1 genomes as early as 15 min and until 60 min post infection, but viral heterochromatin formation was independent of IFI16 [34]. For a more detailed assessment of all cellular DNA-sensing and immune-evasion of herpesviral infection, however, we would like to recommend the excellent reviews by Stempel and colleagues and Orzalli and colleagues, both of which discuss the current knowledge in great detail [35,36].

## 3. DNA Damage Response Proteins

The relationship between herpesviruses and cellular DNA-damage response (DDR) proteins is controversial [37]. While selected DDR proteins are beneficial for viral replication, and are even actively recruited into viral replication compartments at later stages of the lytic cycle, the virus has to antagonize at least parts of the DDR at early stages of infection. Although there are only few reports of early herpesviral antagonism of the DNA damage response, the well-studied DNA viruses from the adenovirus family can serve as an archetypical example. In this regard, adenoviruses have been shown to inhibit several components of the DDR after infection, in particular the non-homologous end-joining (NHEJ) pathway, in order to promote viral replication [38,39,40].

Cellular DDR is composed in a modular way [37]. On top of the cascade are proteins that sense DNA damage event by directly binding to damaged DNA, hallmarked by DNA double strand breaks or single-stranded DNA. The Ku70/Ku80 heterodimer, as well as the Mre11/RAD50/NBS1 (MRN) complex, recognize DNA double strand breaks [41]. In addition, the RPA protein binds to unusual stretches of ssDNA at stalled or stressed replication forks, recruiting ATRIP. After DNA binding, a carboxyterminal motif of one of each sensors (NBS1, ATRIP, KU80 respectively) interacts with its corresponding member of the large phosphatidylinositol-3-kinase like kinases (PI3KKs), ataxia telangiectasia mutated (ATM), ataxia telangiectasia mutated (ATR), and the DNA-dependent protein kinase catalytic subunit (DNA-PKcs), and induces their autophosphorylation and activation [41]. DNA double strand break sensing by the MRN complex leads to the activation of ATM, whereas ssDNA sensing by RPA activates ATR. The activated PI3KKs then phosphorylate the downstream protein H2AX at serine residue 139 (the phosphorylated form is called γH2AX). H2AX is a DNA damage-specific histone variant, and its phosphorylation results in the recruitment of additional factors to the site of DNA damage like RNF8 and RNF168.

The initiation of the DDR cascade is a fast process. All proteins are ubiquitously present in the nucleus, and upon activation, the phosphorylation cascade leads to locally-restricted DDR activation at the point of damage [41]. In addition, there is co-regulation with the cell cycle. From a cellular point of view, the DNA damage must be detected and resolved before the progression of the cell cycle to prevent further mutations and passing of the DNA damage to daughter cells. Therefore, a DDR also results in the activation of cell cycle checkpoints to prevent the replication of damaged DNA. ATM, as well as ATR, phosphorylate checkpoint kinases CHK1 and CHK2, which results in a G1-S-phase checkpoint arrest by activating the tumor suppressor p53.

The herpesviral genome enters the nucleus in its naked and linear form [5]. The viral genome ends resemble DNA double strand breaks (DSB), and in normal cells, a chromosomal DSB is a severe DNA-damage event that is sensed by DDR sensors and repaired immediately. Moreover, it has been demonstrated for HSV-1 that the incoming viral genome contains nicks and gaps that could also activate cellular DDR signaling [42]. Cellular chromosomes are also linear DNA molecules, but their ends are protected by telomeres [43]. Most herpesviral genomes, however, don’t have terminal telomeric repeats or similar structures, and therefore, the viruses have to prevent the initiation of a cellular damage response in order to avoid unwanted repair of their genomes early after infection. Of course, there are exceptions: the genomes of human Herpesviruses-6 (HHV6-A and HHV-6B) and -7 contain human telomere-like sequences [44]. Interestingly, while almost all other herpesviral genomes persist as episomal DNA molecules in infected cells after the establishment of latency, germline-integrated HHV-6 genomes have been reported in approximately 1% of patients. In these, the viral genome is found in telomeric regions, to which it is targeted via its viral telomere sequences [45,46]. As these viruses seem not to yield infectious progeny, the significance of this host genome integration for viral pathogenesis is currently under investigation [44].

It could be demonstrated for all herpesvirus families that specific DDR proteins are activated at later stages of the herpesviral replication cycle during lytic replication. Once viral replication compartments are formed, several DDR proteins are recruited into replication compartments. It has been shown for HSV-1 that ATR and ATRIP are recruited to viral replication compartments, and that ATR pathway proteins are needed for efficient viral replication [47,48,49] while downstream ATR signaling is blocked simultaneously [48,50,51,52]. Excellent work by the Weitzman lab showed that ATM and MRE11 are also activated and needed for efficient viral replication [53,54]. In addition, several cellular DDR proteins, including members of the mismatch repair complex (MSH2, MSH6), the double strand repair protein RAD50, and members of the single-strand repair complex (XRCC1, PARP1), among others, have been demonstrated to associate with viral DNA replication forks by the precipitation of EdC-labeled viral DNA [55], indicating a role of selected DDR proteins during HSV-1 replication. In the case of KSHV, both the Ku70/80 complex, as well as MRN complex, colocalize with viral replication compartments [56] and lytic replication of KSHV activates ATM, DNA-PK, and γH2AX, but not ATR and CHK1 signaling [57]. As mentioned above, DDR proteins are equally distributed in the nucleus, and it has been demonstrated for HCMV that a great number of DDR proteins are activated upon infection, but only a fraction colocalize with viral DNA [58], pointing at a selective regulation of access to viral replication compartments. Therefore, it is important to assess the subnuclear localization of DDR proteins relative to viral DNA to discriminate unspecific global bystander activation and specific local responses. For HCMV, it was further demonstrated that infection results in ATM activation and downstream signaling, leading to the activation of p53 and γH2AX, and that this is required for efficient viral replication [59,60,61,62,63]. Taken together, all members of the alpha-, beta- and gamma-herpesvirus subfamilies have in common that they activate the ATM response at later time points during infection, indicating an important role of ATM activation for viral replication. Furthermore, an additional, unique set of DDR proteins are activated for each individual virus.

**Figure 1:**
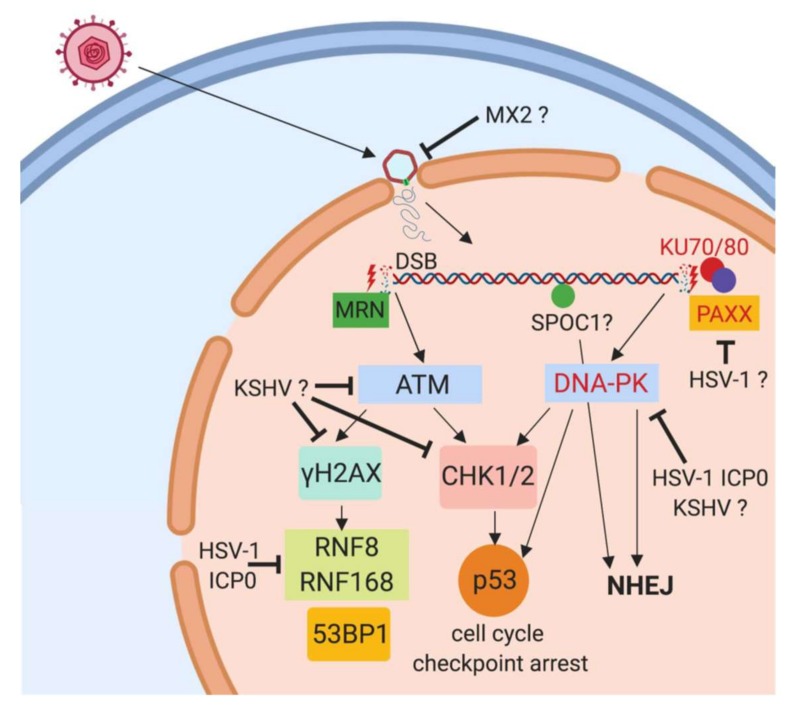
Herpesviral antagonism of the ATM- and DNA-PK-branch of the DDR directly after infection. After infection of target cells, the herpesviral capsid is transported to the nuclear membrane and the viral DNA released into the nucleus through nuclear pores. We propose that the cellular DDR complexes recognize the viral linear DNA by MRN- and KU70/KU80-complex binding to double-strand break resembling ends of the linear viral DNA. This results in activation of kinases ATM and DNA-PK respectively, followed by phosphorylation of H2AX (γH2AX) and CHK1/2 and activation of downstream proteins including the cell cycle regulator p53. HSV-1, HCMV and KSHV have been shown to counteract this activation as indicated in the graph, however, the viral effector proteins and mechanisms are not identified. Depletion of proteins depicted in red font has been shown to enhance herpesviral replication, indicating an inhibiting role on herpesviral replication. ? indicates an unknown mechanism.

However, we hypothesize that the viruses need to block the cellular DDR immediately after infection to prevent the repair of incoming linear genomes. (Figure 1) Herpesviral infection usually takes place in quiescent cells [64]. During the G1- and G0-phase, the NHEJ pathway, activated by KU70/KU80, is the default repair pathway for DSBs. In contrast, the homologous repair (HR) pathway, which is activated by the MRN-complex via ATM, is active during S-phase and G2-phase when sister chromatids are present as a template for homologous recombination. Repair through NHEJ would lead to a loss of genetic information for the virus due to DNA end processing prior to ligation by DNA ligase IV/XRCC4. As mentioned, adenoviruses, a dsDNA virus family that also replicates in the nucleus, have evolved potent mechanisms to block NHEJ. Therefore, we think that herpesviruses also need to block NHEJ in favor of HR in order to achieve the loss of recircularization of their genomes. It was demonstrated that the NHEJ protein PAXX acts as a restriction factor for HSV-1 replication [65]. PAXX interacts with Ku80 and gets relocated after HSV-1 infection, and depletion of PAXX has been shown to enhance HSV-1 replication [65]. For KSHV, the depletion of Ku80 and DNA-PK, two proteins involved in NHEJ, leads to enhanced genome replication [56]. Another important protein in this regard is SPOC1, a cellular chromatin remodeling factor that inhibits immediate early gene expression of HCMV and also adenoviruses [66,67]. Upon DNA damage, SPOC1 is recruited to DNA double strand breaks by ATM signaling, promotes HR-repair, and blocks NHEJ. Moreover, SPOC1 interacts with KAP-1, inhibits KAP-1 phosphorylation, and enhances H3K9 trimethylation [68]. Although SPOC1 showed the unambiguous features of an antiviral restriction factor, surprisingly, it was found to be actively upregulated by the HCMV protein IE-1 shortly after infection [66]. Since SPOC1 restricts both adenoviruses and HCMV, it would be very interesting to see whether SPOC1 also restricts the herpesviruses of the alpha- and gamma-subfamilies.

In general, it is challenging to assess the early events minutes after viral infection, considering that under physiological conditions, only one or very few viral genomes enter the nucleus upon infection, and that one must discriminate between viral and cellular DNA. On these grounds, it is conceivable that data on these early events are partially conflicting. For example, it is textbook knowledge that the incoming linear viral genomes have to undergo recircularization before viral genome replication begins. However, controversial data has been published on whether or not HSV-1 genomes undergo recircularization upon infection [69,70]. In addition, it was assumed that herpesviral genomes were amplified by rolling circle replication during lytic infection of HSV-1, and that HSV-1 proteins were indeed able to induce rolling circle replication in vitro [71,72]. In contrast, HSV-1 replication also leads to replication intermediates that hint at an alternative recombination-based mechanism of replication [73,74,75,76]. Nevertheless, it is beyond doubt that independent of the mode of genome recircularization and viral DNA replication, a loss of genetic information would be detrimental for viral replication. Therefore, it is advantageous for a herpesvirus to prevent NHEJ, and instead, to create an environment that promotes homologous recombination. The ends of linear herpesviral genomes contain at least two but up to 50 repeats (depending on the herpesvirus) that are homologous to each other. These repeats can serve as templates for homologous recombination, and enable genome circularization. From the concatemers formed by rolling circle replication, linear genomes with flanking repeats can reform without losing genetic information, similar to sister chromatids during S-phase. Newer and more sensitive imaging-based detection methods like EdC-/EdU-labeling of viral genomes and fluorescent in situ hybridization-based techniques with single molecule sensitivity could help to resolve these controversies in the future. For example, very elegant work by Jill Dembowski and Neal DeLuca using EdU-labeled viral genomes followed by click-chemistry demonstrated an association of several DDR proteins including 53BP1, PARP1, PARP14, MRE11A, and Ku70(XRCC6) with HSV-1 DNA at early time points post infection [77]. However, one has to consider that EdC/EdU-labeled genomes can be sensed by cellular DNA-damage sensors and induce DDR signaling, which might make an assessment of the results difficult [78,79].

HSV-1 genomes alone are able to induce DNA-PK activation [42]; however, this activation is abrogated upon viral infection by proteasomal degradation of the catalytic subunit of DNA-PK by ICP-0 [80,81]. In addition, HSV-1 replication is drastically enhanced in Ku-deficient cells [82], indicating an inhibitory role of the NHEJ pathway in HSV-1 replication. For HSV-1, it was further demonstrated that both RNF8 and RNF168 are antiviral proteins that are counteracted by HSV-1 protein ICP-0 [83,84]. The viral ubiquitin E3 ligase ICP-0 mediates the proteasomal degradation of RNF8 and RNF168, and thereby, prevents recruitment of downstream proteins like BRCA1 and 53BP1 to γH2AX foci [83,84]. RNF8 and RNF168 have also been shown to mediate ubiquitination of histone H2A, and thereby, induce genome silencing [85].

Taken together, we think that blocking NHEJ immediately after infection and creating a nuclear environment favoring homologous recombination is beneficial for herpesviruses by promoting genome replication and preventing loss of viral genetic information.

## 4. PML Nuclear Bodies

**Figure 2:**
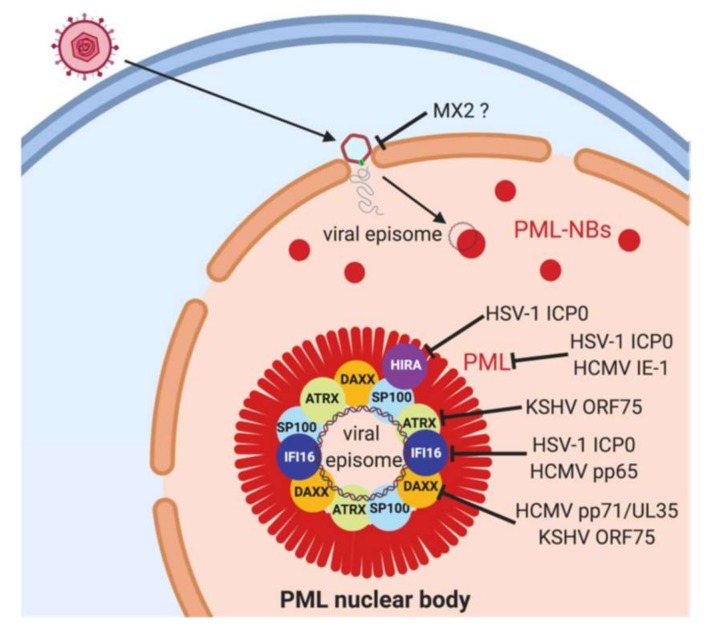
Herpesviral antagonism of PML-nuclear bodies. After the infection of target cells, the herpesviral capsid is transported to the nuclear membrane and the viral DNA released into the nucleus through nuclear pores. The genome rapidly associates with PML nuclear bodies (PML-NBs). PML-NBs are considered as antiviral nuclear organelles that restrict gene expression from viral genomes, and PML-NB components PML, SP100, DAXX and ATRX as well as the transiently associated proteins IFI16 and HIRA have been shown to mediate this restriction. PML is an essential structural component of PML-NBs and depletion of PML disrupts PML-NBs. Herpesviruses in contrast have evolved mechanisms to counteract this restriction, in particular viral tegument and immediate-early proteins as depicted in the graph. ? Indicates hypothetical mode of interference.

After entering the nucleus, herpesviral genomes have been shown to associate with subnuclear structures called PML-nuclear bodies (PML-NBs, or Nuclear Domain 10 (ND10)) [86]. (Figure 2) PML-NBs are named after their key structural component, the promyelocytic leukemia protein (PML or TRIM19). PML is a TRIM protein containing the eponymous tri-partite motif that confers E3-ligase activity. PML is a SUMO-E3 ligase, and accordingly, PML-NBs are described as principal aggregations for SUMOylated proteins in the nucleus. PML-NBs are also involved in other important nuclear functions as storage organelle for nuclear proteins and centers for active transcription. For a detailed overview on the architecture and the multiple functions of PML-NBs, we recommend excellent reviews by others [87]. However, most importantly, PML-NBs are nuclear sites that mediate the antiviral restriction of DNA-virus gene expression and replication [88,89]. Several PML-NB components like PML itself, SP100, DAXX, and ATRX are bona fide restriction factors for viral infection, undermined by the observation that PML-NB components like PML, Sp100, and DAXX are induced by interferon [90]. DAXX and ATRX form a histone chaperone complex that recruits the non-canonical histone variant H3.3 to incoming viral genomes, as has been reported for the adenoviruses, HCMV and EBV [91,92,93]. In the case of HSV-1, H3.3 is also recruited by the histone chaperone HIRA [94,95]. Moreover, DAXX and ATRX interact with epigenetic modifiers like HP1 and histone methyl-transferase EZH2. It is thought that thereby a repressive chromatin environment is generated that mediates the epigenetic silencing of incoming viral genomes, and thus hampers viral gene expression and replication. For HSV-1, it was demonstrated that viral chromatin is established on the incoming genome as early as 1–2 h post infection [96]. Interestingly, predominantly repressive histone modifications like H3-lysine 9-tri-methylation (H3K9me3) and H3-lysine 27 trimethylation (H3K27me3) can be found on viral chromatin, which prevents viral gene expression and replication [96]. As mentioned above, ATRX, PML, and IFI16 are associated with the HSV-1 genome within the first hour after infection, and initial heterochromatin formation is dependent on ATRX [34]. For HCMV, viral genomes become associated with PML after infection, and similar to HSV-1, expression from the major immediate-early promoter (MIEP) is also repressed through histone modification of the region [97,98,99].

PML is the key protein of PML-NBs that forms a lattice and serves as a docking site for other PML-NB components, and the degradation of PML leads to a dispersal of all PML-NB proteins. Studies by Gerd Maul and colleagues have suggested that PML-NBs are needed for efficient viral gene expression [100]; meanwhile, it is well accepted in the field that PML-NBs reflect an antiviral entity in the nucleus, which is also supported by several hundred publications encompassing almost all DNA viruses [87,90].

The viruses in turn have evolved mechanisms to antagonize restriction by PML-NB. (Figure 2) In this review, we focus on the early events after infection, and thus, on the tegument proteins of HSV-1, HCMV, and KSHV; for herpesviral proteins that are de novo expressed at immediate, early, and later time points, and that antagonize PML-NBs, we recommend reviews by others [89,101]. In the case of HSV-1, the proteins VP16 and ICP0 facilitate viral gene expression and the removal of viral heterochromatin [102,103]. ICP0 was one of first viral proteins discovered to antagonize PML [104]. ICP-0 is an immediate early protein of HSV-1, and also part of the virus particle. It is a ubiquitin E3 ligase that mediates the ubiquitin-mediated degradation of target proteins, including PML [105]. An HSV-1 ICP-0 deficient virus, as well as HCMV, was restricted by PML, DAXX, and Sp100 in a cooperative manner, shown by shRNA depletion of individual proteins [106]. The replication of the ICP-0 deficient virus was affected at different replication steps [107], further corroborating the importance of PML-NB proteins on viral replication and also the role of ICP-0 in antagonizing PML-NBs. As mentioned, PML-NBs are linked to SUMOylation, and interaction between PML-NB components heavily depends on interaction between SUMOylated proteins and SUMO-interaction motif (SIM-) containing proteins [87]. Accordingly, herpesviruses have also evolved ways to target the SUMOylation of proteins in general to antagonize PML-NB function. Excellent work by the Everett lab demonstrated that ICP-0 acts as a SUMO-targeted ubiquitin ligase (STUbL) which recognizes SUMOylated proteins and ubiquitinates them [108]. K48-linked ubiquitination of SUMOylated proteins by ICP-0 leads to the subsequent proteasomal degradation of target proteins [108].

In the case of HCMV, two proteins have been shown to be of particular importance for PML-NB antagonism: the tegument protein pp71 and the immediate-early protein 1 (IE-1). pp71 enters the nucleus together with the viral genome and induces the degradation of DAXX and the sequestration of ATRX from PML-NBs [109,110]. This first line of PML-NB antagonism enables the expression of immediate-early proteins including IE-1. IE-1 in turn efficiently dissolves PML-NBs [111], in particular by interfering with the de novo SUMOylation of PML [112], and thereby enables the expression of early genes. Most protein–protein interactions in PML-NBs are mediated by SUMOylation; therefore, SUMOylation of PML is essential for the formation of PML-NBs [87].

All gammaherpesviruses like KSHV encode for at least one member of a viral protein family, the viral formylglycineamide ribonucleotide amidotransferase (vFGARAT) [89]. The vFGARATs are cellular FGARAT (also PFAS, EC 6.3.5.3) homologs, the enzyme that catalyzes the fourth step in the de novo purine biosynthesis pathway. Several family members have been shown to counteract the antiviral function of PML-NBs since the homology of the viral and the cellular gene were first described in 1997 [89,113]. In doing so, different gammaherpesviruses antagonize different components of PML-NBs. The vFGARAT of KSHV for example, ORF75, induces the degradation of ATRX and the relocalization of DAXX [114], whereas closely related human and monkey viruses apply different strategies [89,93,115,116,117]. vFGARATs are tegument proteins that enter the newly-infected cell together with the viral genome. Similar to ICP-0 of HSV-1 and pp71 of HCMV, it is thought that the immediate antagonism of PML-NBs upon entering of the nucleus is needed for efficient viral early gene expression. In addition, vFGARATs apparently have additional functions needed for viral infection and replication, including structural functions as tegument components and involvement in deamidation of target proteins [89,118,119,120]. This is also reflected by genetic depletion of ORF75 from the KSHV genome, which results in a replication-dead virus that cannot be explained solely by its effect on PML-NBs [114].

Intriguingly, the discussed pathways seem to be interconnected in several ways. It is established that upon initiation of a DDR, PML-NB numbers per cell increase and PML-NBs are recruited to sites of DNA damage. The PML-NB components DAXX and ATRX for example are important antagonists of the alternative lengthening of telomeres- (ALT-) pathway [121]. ALT is a recombination-based mechanism of telomere maintenance that gets activated during early development, and in a subset of cancer cells. One could speculate that the inactivation of ATRX and DAXX activates ALT (or other recombination-based pathways), and thereby contributes to recombination of the viral genome [122]. IFI16, the cellular sensor for dsDNA has, in turn, been shown to associate with PML-NBs, although the antiviral mechanisms by which PML-NB components like PML and ATRX restrict HSV-1 seem to be different, and not directly connected to IFI16 [20,123]. Along this line, a novel connection between IFI16, the DDR kinase ATM, and the cellular DNA-sensing pathway molecule STING has been demonstrated recently [124]. Upon DNA-damage, IFI16, ATM, and PARP1 form a complex that leads to non-canonical STING activation via TRAF6 activation, resulting in NF-kB activation [124]. In addition, the DDR proteins RNF8 and RNF168 have been shown to be negative regulators of PML [125]. An interesting viral protein that has been shown to alter both PML-NBs and the DDR is pUL35 of HCMV. pUL35 is part of the virion, interacts with pp71, and activates the HCMV MIEP together with pp71 [126]. The expression of pUL35 leads to formation of UL35 nuclear bodies within the nucleus [127]. Those bodies form independently of PML, but after the formation PML, Sp100, and DAXX, get recruited to UL35 bodies [127]. In addition, pUL35 has also been shown to activate the DDR by inducing 53BP1 and γH2AX foci [128].

Beside the cellular DNA sensors, DDR proteins and PML-NB components, there are a variety of other cellular proteins that have been demonstrated to inhibit herpesviral immediate-early gene expression, especially proteins involved in epigenetic regulation and chromatin remodeling. For example, in addition to its well-known role in the restriction of HIV, it was recently recognized that the human interferon induced dynamin-like GTPase MX2 (MxB) interferes with HSV-1 entry at a step after tegument dissociation but before viral genome uncoating and translocation into the nucleus [129,130,131,132,133]. Although the detailed mechanism of this restriction is not fully elucidated, it is clear that it requires the GTPase function of MX2.

Taken together, we think that herpesviruses have to counteract several cellular pathways to enable viral gene expression and prevent silencing of the viral genome immediately after infection. Specifically, cellular sensors of herpesviral DNA, DNA-damage proteins, and components of PML-nuclear bodies are described as antiviral restriction factors. In turn, members of all herpesvirus subfamilies have evolved efficient countermeasures to evade cellular intrinsic immunity, in particular by viral tegument proteins that enter the nucleus together with the viral genome. However further studies are needed to analyze this viral and cellular arms race in order to get more insights that might help to identify new targets for antiviral drugs that could be used for therapy of patients in the future.
